# Primary 1,25-Dihydroxyvitamin D_3_ Response of the Interleukin 8 Gene Cluster in Human Monocyte- and Macrophage-Like Cells

**DOI:** 10.1371/journal.pone.0078170

**Published:** 2013-10-21

**Authors:** Jussi Ryynänen, Carsten Carlberg

**Affiliations:** School of Medicine, Institute of Biomedicine, University of Eastern Finland, Kuopio, Finland; University of Oxford, United Kingdom

## Abstract

Genome-wide analysis of vitamin D receptor (VDR) binding sites in THP-1 human monocyte-like cells highlighted the interleukin 8 gene, also known as chemokine CXC motif ligand 8 (*CXCL8*). CXCL8 is a chemotactic cytokine with important functions during acute inflammation as well as in the context of various cancers. The nine genes of the CXCL cluster and the strong VDR binding site close to the *CXCL8* gene are insulated from neighboring genes by CCCTC-binding factor (CTCF) binding sites. Only *CXCL8*, *CXCL6* and *CXCL1* are expressed in THP-1 cells, but all three are up-regulated primary 1,25-dihydroxyvitamin D_3_ (1,25(OH)_2_D_3_) target genes. Formaldehyde-assisted isolation of regulatory elements sequencing analysis of the whole CXCL cluster demonstrated 1,25(OH)_2_D_3_-dependent chromatin opening exclusively for the VDR binding site. In differentiated THP-1 cells the *CXCL8* gene showed a 33-fold higher basal expression, but is together with *CXCL6* and *CXCL1* still a primary 1,25(OH)_2_D_3_ target under the control of the same genomic VDR binding site. In summary, both in undifferentiated and differentiated THP-1 cells the genes *CXCL8*, *CXCL6* and *CXCL1* are under the primary control of 1,25(OH)_2_D_3_ and its receptor VDR. Our observation provides further evidence for the immune-related functions of vitamin D.

## Introduction

Chemokines are small (8-11 kDa), pro-inflammatory cytokines that are involved in trafficking, activation and proliferation of many cell types, such as myeloid, lymphoid, epidermal and endothelial cells [1]. The over 50 presently known chemokines have been assigned according to the arrangement of their conserved cysteine motifs into the four classes C, CC, CXC and CX3C [2-4]. Chemokine CXC motif ligand (CXCL) 8, also known as interleukin 8, is the first chemokine discovered some 25 years ago [5] and a prototypical member of the CXC chemokine family. CXCL8 is one of the most potent neutrophil chemo-attractants in acute inflammation [6], i.e. it is among the first signals to be expressed and released by the various cell types involved in acute inflammation. CXCL8 binds with similar high affinity to the G-protein-coupled receptors CXCR1 and CXCR2 [7,8] and initiates downstream signaling, such as the regulation of fibroblast growth factor 2 and androgen receptor [9,10], suggesting that CXCL8 is also implicated in the control of cellular proliferation, such as in benign prostate hyperplasia and prostate cancer.

The genes encoding for CXCLs 1-8 form together with a variant of *CXCL4*, *CXCL4.1* [11], a cluster of nine neighboring genes spanning over 350 kb of chromosome 4. Since chemokines are not stored intracellularly but secreted dependent on a stimulus, their effects rely on transcriptional regulation and *de novo* protein synthesis. The transcriptional regulation of the whole CXCL cluster is largely elusive, but the transcription factors nuclear factor kappa-light-chain-enhancer of activated B cells (NF-κB) and activator protein 1 are known to control *CXCL8* and *CXCL1* gene transcription [12-14].

The active compound of the vitamin D endocrine system, 1,25-dihydroxyvitamin D_3_ (1,25(OH)_2_D_3_), is not only involved in calcium and phosphate homeostasis and bone mineralization [15], but there is both epidemiological and pre-clinical evidence that 1,25(OH)_2_D_3_ also has anti-proliferative and immuno-modulatory functions [16,17]. In the context of the latter, it had been reported that in immune-stimulated monocytes 1,25(OH)_2_D_3_ is able to reduce the interferon γ-mediated up-regulation of the mRNA expression of the cytokines tumor necrosis factor α, interleukin 6 and 1 and of *CXCL8* over a time-span of 48 h [18]. In fact, 1,25(OH)_2_D_3_ has also been shown in other studies to counteract to pro-inflammatory signal transduction pathways, such as NF-κB signaling [19], and has specifically shown to inhibit the NF-κB-mediated up-regulation of *CXCL8* [20].

1,25(OH)_2_D_3_ is a nuclear hormone that binds directly to the transcription factor vitamin D receptor (VDR) [21], which is a member of the nuclear receptor superfamily [22]. VDR, like most other transcription factors, competes with the intrinsic repressive nature of chromatin for access to its genomic binding sites [23,24]. Already in the absence of ligand VDR is able to contact genomic DNA and then preferentially forms complexes with co-repressor proteins [25] and chromatin modifying enzymes, such as histone deacetylases (HDACs) [26]. However, in the presence of ligand VDR interacts with co-activator proteins and histone acetyltransferases [27]. Therefore, interaction with chromatin and its modifying enzymes is a central element in 1,25(OH)_2_D_3_ signaling [28]. A direct modulation of transcription by 1,25(OH)_2_D_3_ through the interaction of activated VDR with the basal transcriptional machinery is achieved via the specific association of VDR with its genomic binding sites. VDR binding sites, referred to as response elements, are preferentially formed of a direct repeat of two hexameric binding motif spaced by three nucleotides (DR3) [29,30]. Within the last three years the genome-wide binding of VDR has been determined by chromatin immunoprecipitation (ChIP) coupled with massive parallel sequencing (ChIP-seq) in human lymphoblastoid cells (treated for 36 h with 1,25(OH)_2_D_3_ [31]), in human monocyte-like cells (undifferentiated THP-1, stimulated for 40 min with 1,25(OH)_2_D_3_ [32]), in human colorectal cells (LS180, exposed for 180 min with ligand [33]) and in human hepatic stellate cells (LX2, incubated for 16 h with the 1,25(OH)_2_D_3_ analog MC903 [34]). These four studies revealed 1,600-6,200 specific VDR binding sites, but only a low percentage of them are identical in all investigated cellular models [35]. Moreover, only approximately 30% of these VDR binding sites carry a DR3-type sequence that has a high similarity score with the consensus sequence. This suggests that there are additional modes of VDR binding to its genomic targets [36].

In earlier studies [32,37,38] we have demonstrated that THP-1 cells represent a well responding and physiologically meaningful model system for the investigation of 1,25(OH)_2_D_3_ signaling in the context of innate immunity and cancer. In this study, we investigated the response of *CXCL8* and other members of the CXCL cluster in undifferentiated THP-1 cells (monocyte-like cells) and phorbol 12-myristate 13-acetate (PMA)-differentiated THP-1 cells (M2-type macrophage-like cells). We found that in both forms of THP-1 cells the neighboring genes *CXCL8*, *CXCL6* and *CXCL1* are primary 1,25(OH)_2_D_3_ targets being controlled by the same genomic VDR binding site. This provides further evidence for the immune-related functions of vitamin D.

## Material and Methods

### Cell culture

The human acute monocytic leukemia cell line THP-1 [39] was grown in RPMI 1640 medium supplemented with 10% fetal calf serum (FCS), 2 mM L-glutamine, 0.1 mg/ml streptomycin and 100 U/ml penicillin and the cells were kept at 37 °C in a humidified 95% air / 5% CO_2_ incubator. Prior to mRNA or chromatin extraction, undifferentiated THP-1 cells were grown overnight in a density of 500,000 cells/ml in phenol red-free RPMI 1640 medium supplemented with 5% charcoal-stripped FCS. For the differentiation into M2-type macrophage-like cells, THP-1 cells were grown 72 h in normal culture medium supplemented with 20 nM PMA (Sigma-Aldrich). In 1,25(OH)_2_D_3_ stimulation experiments, cells were treated with 10 nM 1,25(OH)_2_D_3_ (Sigma-Aldrich) or solvent (0.001% ethanol). In HDAC inhibition experiments, cells were stimulated with 300 nM TsA, 2 µM suberoylanilide hydroxamic acid (SAHA), 1 mM valproic acid (VPA, all compounds from Sigma-Aldrich), 100 nM 1,25(OH)_2_D_3_ or solvent (0.16% ethanol or 0.02% DMSO).

### RNA extraction, cDNA synthesis and PCR

Total RNA extraction, cDNA synthesis and qPCR were performed as described previously [40]. qPCR reactions were performed with the LightCycler^®^ 480 System (Roche) using 400 nM of reverse and forward primers, 2 to 4 µl cDNA or ChIP template and the LightCycler 480 SYBRGreen I Master mix (Roche) or the Maxima^TM^ SYBR Green/ROX qPCR Master mix (Fermentas). Primer-specific temperatures are listed in Tables S1 and S2. Relative mRNA expression levels were determined using the formula 2^-(ΔCt)^, where ΔCt is Ct_(target gene)_ – Ct_(reference gene)_. Like in previous studies on interleukin gene expression in THP-1 cells [37,38], in most experiments *RPLP0* was used as a reference gene for normalization. However, in HDAC inhibition experiments, where more global effects on gene expression were anticipated, the target genes were normalized to the three reference genes *B2M*, *GAPDH* and *HPRT1* as described previously [41].

### ChIP-seq and ChIA-PET data visualization

Publically available CTCF ChIP-seq datasets of the ENCODE consortium [42] were downloaded from UCSC (http://genome.ucsc.edu/ENCODE) for K562 human monocytic leukemia cells (wgEncodeEH002279), HUVEC human endothelial cells (wgEncodeEH000054) and NHEK human epidermal keratinocytes (wgEncodeEH000063). Our own VDR ChIP-seq (GSE27437) and FAIRE-seq datasets (GSE40075) are available at GEO (www.ncbi.nlm.nih.gov/geo). The Integrative Genomics Viewer (IGV) [43] was used to visualize ChIP-seq and FAIRE-seq data. The chromatin interaction analysis with paired-end tag sequencing (ChIA-PET) data for CTCF-mediated chromatin loops in K562 cells (wgEncodeEH002075) was visualized using the UCSC genome browser (http://genome.ucsc.edu) [44].

### ChIP

ChIP was performed as described previously [40]. After 10 min crosslinking, undifferentiated THP-1 cells were collected by centrifugation, while adherent PMA-differentiated THP-1 cells were scraped into Farnham Lysis buffer (0.5% NP-40, 85 mM KCl, protease inhibitors, 5 mM PIPES, pH 8.0) and then pelleted. Immunoprecipitation was carried out by using 1 µg of anti-VDR antibody (sc-1008, Santa Cruz Biotechnologies), CTCF antibody (12-309, Millipore) or non-specific IgG (12-370, Millipore), which were pre-bound to 20 µl Magna ChIP™ Protein A Magnetic Beads (Millipore). Before DNA isolation, samples were reverse cross-linked at 65 °C for 5 h in the presence of proteinase K (Roche) in a final concentration of 100 µg/ml. Equal DNA amounts of chromatin fragments, measured with Quant-iT™ PicoGreen^®^ dsDNA Assay Kit (Invitrogen), were analyzed by qPCR.

## Results

### The CXCL gene cluster

**Figure 1:**
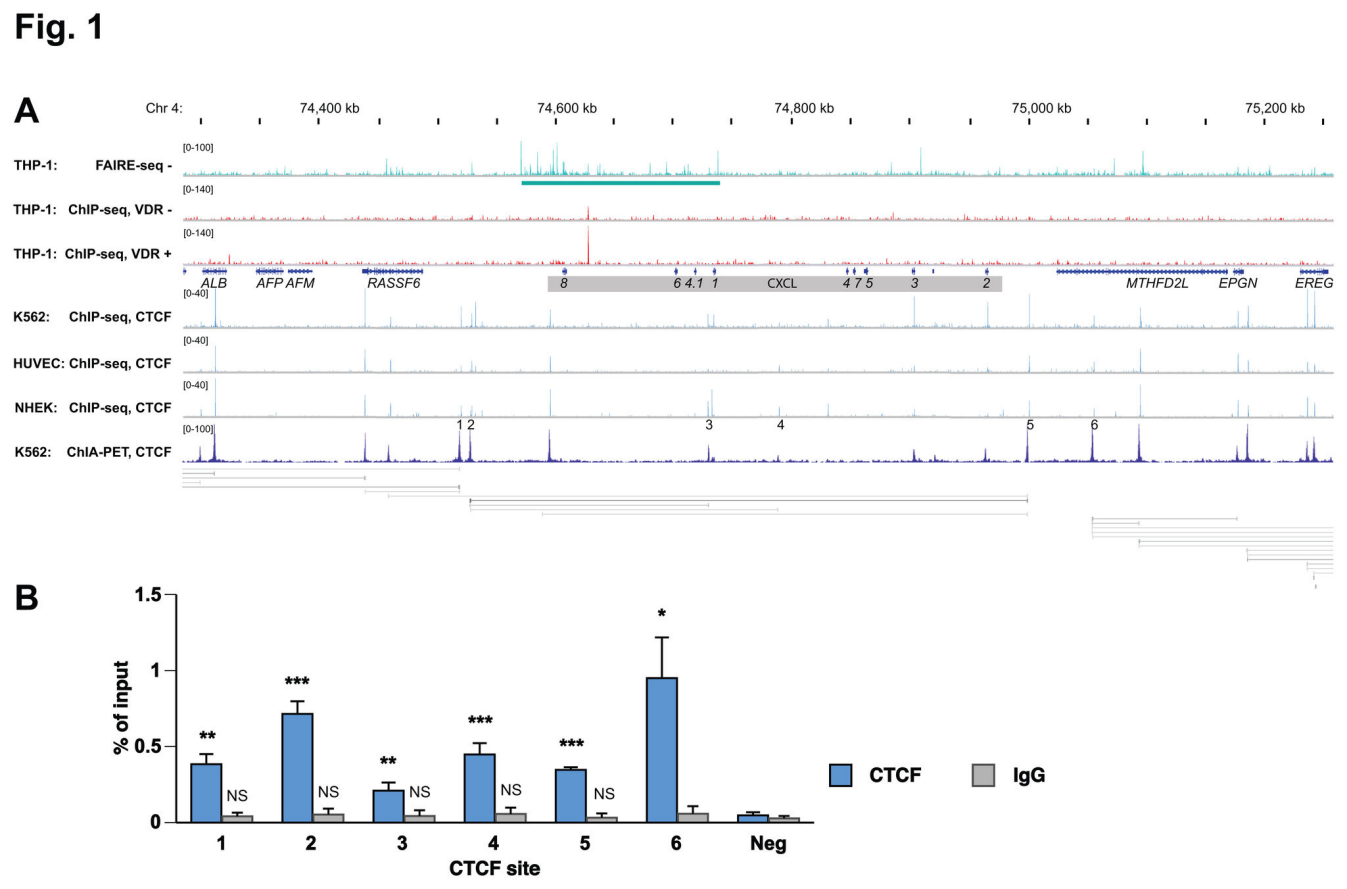
Genome view of the CXCL gene cluster. A. The IGV browser was used to show the peak tracks of FAIRE-seq data from THP-1 cells [51] (stimulated for 20 min with ethanol, turquoise) and VDR ChIP-seq data from THP-1 cells [32] (unstimulated (-) and treated for 40 min with 1,25(OH)_2_D_3_ (+), red). The gene structures are shown in blue and the 9 genes of the CXCL gene cluster are underlayed in grey. The THP-1 data were compared with CTCF ChIP-seq data from the ENCODE cell lines K562, HUVEC and NHEK [42] (blue) and CTCF ChIA-PET data [47] in track view (dark blue) and in looping view (grey horizontal lines). Six conserved CTCF sites were highlighted. B. ChIP-qPCR was performed with chromatin samples obtained from unstimulated THP-1 cells to determine CTCF (blue) and unspecific IgG (grey) binding at the six genomic regions, which were suggested by data obtained in K562 cells (see panel A). Columns represent the means of at least three independent experiments and the bars indicate standard deviations. Two-tailed Student’s t-tests were performed to determine the significance CTCF association in reference to a control region from chromosome 6 (* p < 0.05; ** p < 0.01; *** p  < 0.001).

Formaldehyde-assisted isolation of regulatory elements sequencing (FAIRE-seq) is a method that allows the identification of chromatin sites devoid of nucleosomes, roughly translating to the genome-wide localization of chromatin regions that are accessible to transcription factors, such as VDR, at a given time and condition [48,49]. In this study, we used a FAIRE-seq dataset obtained from THP-1 human monocytic leukemia cells [50,51] and aligned the resulting peaks with the VDR ChIP-seq dataset from the same cell line [32]. Interestingly, a chromatin region spanning over 180 kb (from 45 kb upstream of the *CXCL8* gene to 9 kb downstream of the *CXCL1* gene, underlined in the top lane of Figure 1A) displayed a higher rate of open chromatin, since it showed stronger FAIRE signals than its up- and downstream flanking regions. VDR ChIP-seq analysis in THP-1 cells [32] of the same genomic region around the CYCL cluster highlighted a prominent, 1,25(OH)_2_D_3_-inducible VDR binding site 22 kb downstream of the transcription start site (TSS) of the *CXCL8* gene (Figure 1A).

The sequence-specific transcription factor CTCF is known as a chromatin organizer, which links chromosomal domains [45]. Therefore, when neighboring genes are co-regulated, they should to be part of the same chromosomal domain and not be separated by insulating CTCF binding sites. Since CTCF binding sites are highly conserved between tissues and cell lines [46], we used the publically available ChIP-seq datasets of the ENCODE consortium [42] and displayed CTCF binding sites from K562 human monocytic leukemia cells, HUVEC human endothelial cells and NHEK human epidermal keratinocytes over the whole CXCL cluster and its upstream and downstream flanking genes (Figure 1A). A genome-wide map of the 3-dimensional interactions of CTCF in K562 cells, as obtained by ChIA-PET assays [47], was displayed by using the UCSC genome browser (Figure 1A). It demonstrated that the chromosomal domain containing the whole CXCL gene cluster, but not any other genes, spans from a region upstream of CTCF binding site 2 to site 5, i.e. over nearly 500 kb. Moreover, a number of loops connect CTCF site 1 to upstream and CTCF site 6 a to downstream CTCF sites, respectively, i.e. the genes that are located in these flanking regions seem to be part of different chromosomal domains than that of the isolated CXCL gene cluster.

In order to confirm that the CTCF binding sites, which are suggested by ENCODE data, are also used in our THP-1 cell model, we performed ChIP-qPCR with chromatin samples from non-stimulated, undifferentiated THP-1 cells (Figure 1B). In comparison to a negative control region from chromosome 6, we found to all six genomic regions CTCF binding. This means that also in THP-1 cells these conserved CTCF sites are occupied with protein.

In summary, the cluster of nine CXCL genes carries a prominent VDR binding site close to the *CXCL8* gene and is flanked by conserved CTCF binding sites, which define the borders of the chromosomal domain of the gene cluster.

### 1,25(OH)_2_D_3_ response of CXCL cluster genes in undifferentiated THP-1 cells

**Figure 2:**
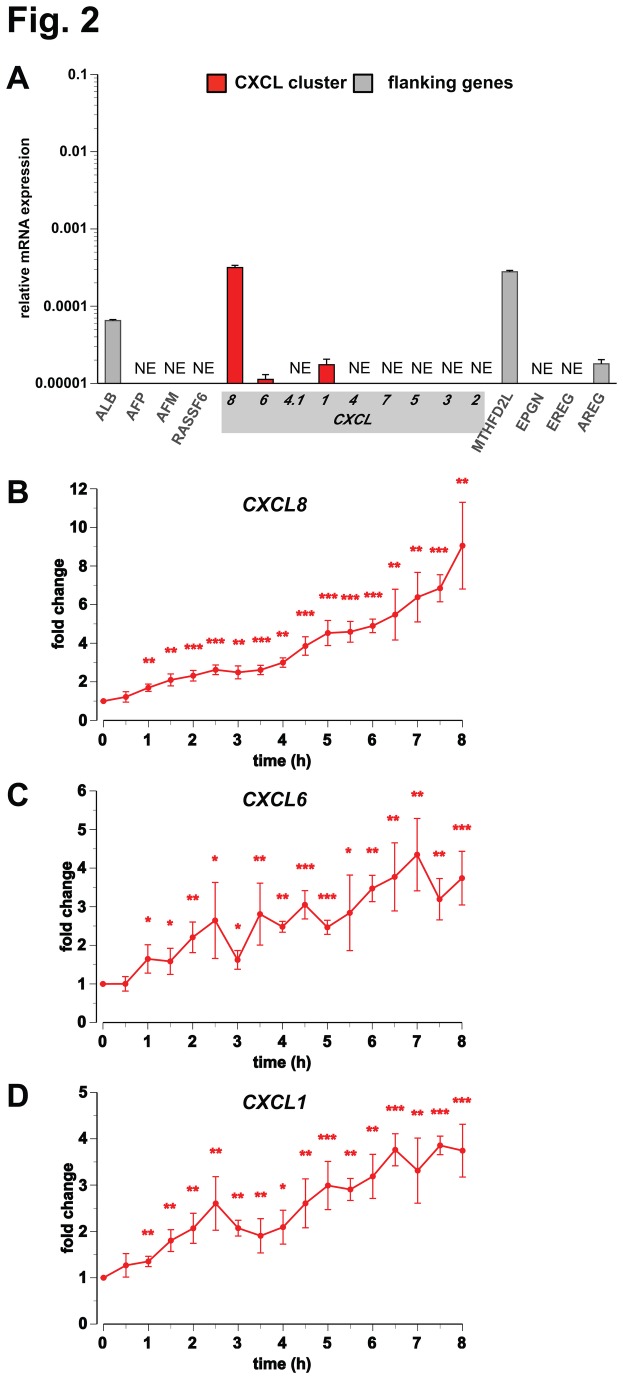
Primary 1,25(OH)_2_D_3_ target genes of the CXCL gene cluster in undifferentiated THP-1 cells. With samples obtained from THP-1 cells qPCR was performed to determine the basal expression, relative to the housekeeping gene *RPLP0*, of the nine genes of the CXCL gene cluster and each four flanking genes (A) and the change of expression of *CXCL8* (B), *CXCL6* (C) and *CXCL1* (D) in response to incubation with 10 nM 1,25(OH)_2_D_3_ over a time period of 8 h. Columns (A) and data points (B-D) represent the means of at least three independent experiments and the bars indicate standard deviations. Two-tailed Student’s t-tests were performed to determine the significance of the mRNA induction by the stimuli (* p < 0.05; ** p < 0.01; *** p < 0.001).

In order to get an overview on the relative basal expression of the members of the CXCL cluster and their upstream and downstream flanking genes, we performed qPCR in non-stimulated, undifferentiated THP-1 cells (Figure 2A). Within the CXCL cluster we could detect the expression of only *CXCL8*, *CXCL6* and *CXCL1*: *CXCL8* is 28- and 18-times higher expressed than *CXCL6* and *CXCL1*, respectively. In addition, from the upstream flanking genes of the CXCL cluster the albumin (*ALB*) gene and from the downstream flanking the genes methylenetetrahydrofolate dehydrogenase (NADP^+^-dependent) 2-like (*MTHFD2L*) and amphiregulin (*AREG*) are expressed in undifferentiated THP-1 cells. Next we stimulated the cells with 1,25(OH)_2_D_3_ and performed qPCR for the six expressed genes, in order to evaluate their possible primary response to the VDR ligand. Interestingly, the detailed time courses indicated that *CXCL8* (Figure 2B), *CXCL6* (Figure 2C) and *CXCL1* (Figure 2D) are already significantly up-regulated 1 h after onset of stimulation with 1,25(OH)_2_D_3_ and reach after 8 h an induction of 9.1-fold for *CXCL8*, 3.7-fold for both *CXCL6* and *CXCL1*, respectively. In contrast, the flanking genes *ALB*, *MTHFD2L* and *AREG* display no significant response to 1,25(OH)_2_D_3_ (data not shown).

Taken together, in undifferentiated THP-1 cells only the CXCL cluster genes *CXCL8*, *CXCL6* and *CXCL1* are expressed, but all three are primary 1,25(OH)_2_D_3_ targets. From the genes flanking the CXCL cluster only *ALB*, *MTHFD2L* and *AREG* are expressed, but none of them responds to 1,25(OH)_2_D_3_ stimulation. 

### Open chromatin within the CXCL cluster

Open chromatin is in general more sensitive to HDAC inhibitors than closed chromatin. Therefore, we assessed *CXCL8*, *CXCL6* and *CXCL1* gene expression after inhibition of HDACs by TsA, SAHA and VPA for 2.5 and 24 h alone and in combination with 1,25(OH)_2_D_3_ (Figure S1). After short-term HDAC inhibitor treatment all three genes were down-regulated: *CXCL8* by VPA, *CXCL6* by SAHA and *CXCL1* by TsA. In contrast, after 24 h CXCL8 was up-regulated by SAHA, *CXCL6* even by both SAHA and VPA, while *CXCL1* showed no response. The 2.5 h treatment with 1,25(OH)_2_D_3_ resulted for all three genes in an approximately 2-fold up-regulation, which is consistent with our time course data (Figure 2). Furthermore, the 24 h time point indicated a prominent long-term stimulation of all three genes by 1,25(OH)_2_D_3_: 32-fold for *CXCL8*, 17-fold for *CXCL6* and 14-fold for *CXCL1*. Consistent with our previous findings [41], at short-term treatment (2.5 h) with 1,25(OH)_2_D_3_ together with TsA, SAHA or VPA the HDAC inhibitors dominated over the VDR ligand. At long-term double treatment (24 h), TsA and SAHA significantly reduced the strong 1,25(OH)_2_D_3_ up-regulation of *CXCL8* and SAHA and VPA that of *CXCL1*. However, the HDAC inhibitors had no significant effect on the 1,25(OH)_2_D_3_ response of the *CXCL6* gene. 

**Figure 3:**
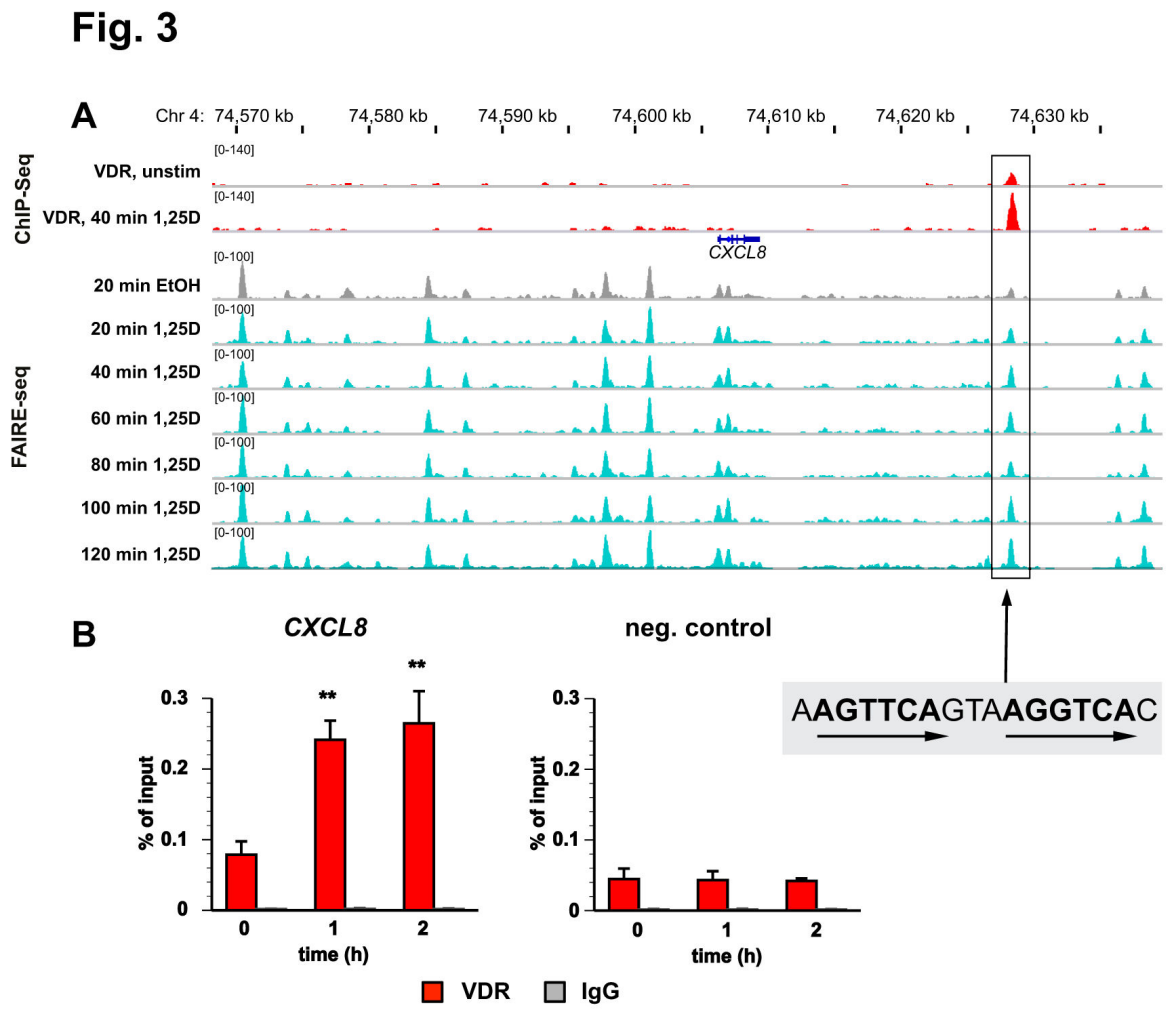
Detailed genomic view of VDR association and 1,25(OH)_2_D_3_-dependent chromatin opening. A. The IGV browser was used to display the genomic region around the *CXCL8* gene. The peak tracks show VDR ChIP-seq data (red [32]) and FAIRE-seq data (grey for the ethanol-treated control, turquoise for the samples treated with 1,25(OH)_2_D_3_ for indicated times [51]), both from THP-1 cells. The gene structures are shown in blue. The sequence of a DR3-type VDR binding site below the summit of the VDR ChIP-seq peaks is indicated. B. ChIP-qPCR was performed with chromatin samples obtained from THP-1 cells to determine VDR association (red) and unspecific IgG binding (grey) at the VDR binding sites close to the *CXCL8* gene and a negative control region of chromosome 6. Cells were stimulated for 0, 1 and 2 h with 10 nM 1,25(OH)_2_D_3_ and chromatin was extracted. Columns represent the means of at least three independent experiments and the bars indicate standard deviations. Two-tailed Student’s t-tests were performed to determine the significance of 1,25(OH)_2_D_3_-induced VDR association in reference to untreated cells (** p < 0.01).

The FAIRE-seq pattern of the genomic region around the genes *CXCL8*, *CXCL6* and *CXCL1* suggests that a treatment with 1,25(OH)_2_D_3_ has no global effect on the number or intensity of sites of open chromatin in THP-1 cells (Figure 1A and data not shown). However, we observed at the VDR binding site close to the *CXCL8* gene a significant, 1,25(OH)_2_D_3_-dependent opening of chromatin in a FAIRE-seq time course experiment with measurements every 20 min over a time period of 120 min (Figure 3A). In order to confirm VDR binding to this site, we performed ChIP-qPCR with chromatin samples from THP-1 cells that had been treated for 0, 1 and 2 h with 1,25(OH)_2_D_3_ (Figure 3B). In comparison to a negative control region from chromosome 6, we observed already in the absence of ligand VDR binding to the site, which significantly increased by the addition of 1,25(OH)_2_D_3_. From previous studies [32,50,51] we know that VDR binding sites at regions of 1,25(OH)_2_D_3_-dependent chromatin opening have genome-wide the highest rate of DR3-type response elements (66%) below VDR ChIP-seq summits. Consistent with this, the VDR binding site close to the *CXCL8* gene also contained a sequence with a high similarity score to a DR3-type response element (Figure 3A).

In summary, the genes *CXCL8*, *CXCL6* and *CXCL1* are sensitive to HDAC inhibitor treatment, which also modulates their response to 1,25(OH)_2_D_3_. The VDR binding site close to the *CXCL8* gene co-locates with a region of 1,25(OH)_2_D_3_-sensitve open chromatin and carries a DR3-type response element.

### 1,25(OH)_2_D_3_ response of CXCL cluster genes in PMA-differentiated THP-1 cells

**Figure 4:**
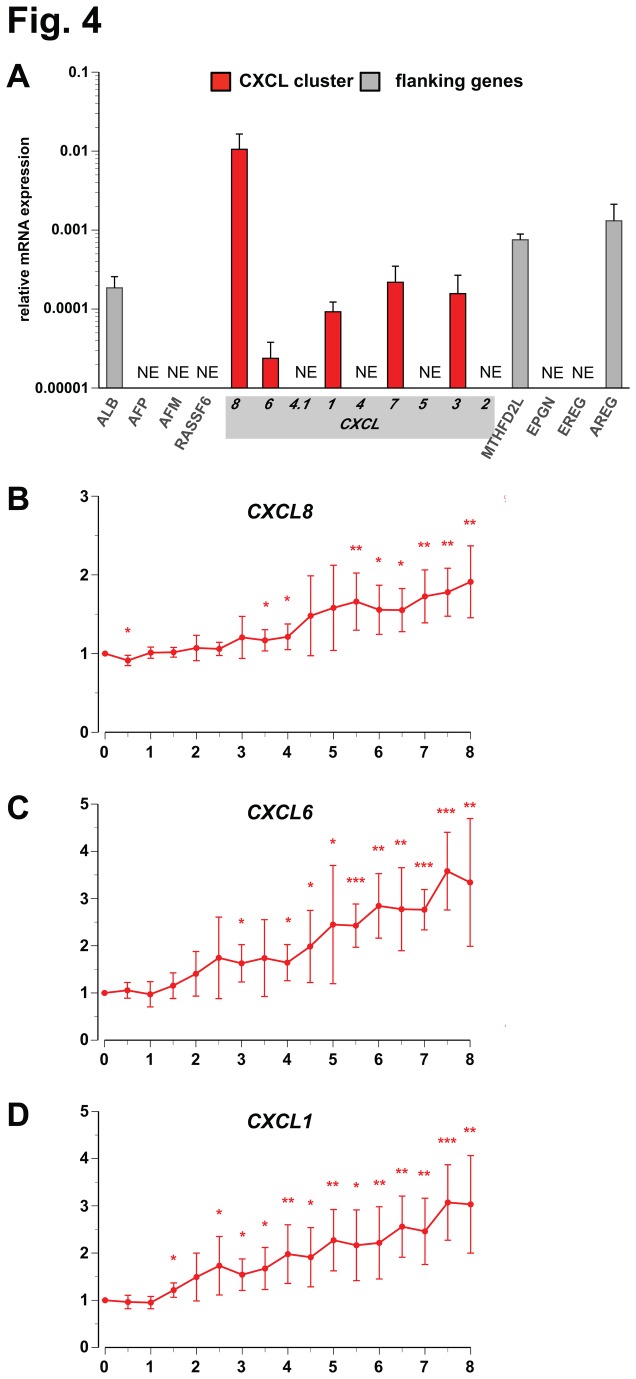
Primary 1,25(OH)_2_D_3_ target genes of the CXCL gene cluster in PMA-differentiated THP-1 cells. With samples obtained from PMA-differentiated THP-1 cells qPCR was performed to determine the basal expression, relative to the housekeeping gene *RPLP0*, of the nine genes of the CXCL gene cluster and each four flanking genes (A) and the change of expression of *CXCL8* (B), *CXCL6* (C) and *CXCL1* (D) in response to incubation with 10 nM 1,25(OH)_2_D_3_ over a time period of 8 h. Columns (A) and data points (B-D) represent the means of at least three independent experiments and the bars indicate standard deviations. Two-tailed Student’s t-tests were performed to determine the significance of the mRNA induction by 1,25(OH)_2_D_3_ (* p < 0.05; ** p < 0.01; *** p < 0.001).

The phorbol ester PMA is known to differentiate in suspension growing THP-1 cells into adherent M2-type macrophage-like cells [52]. In such PMA-differentiated THP-1 cells we used qPCR to compare the basal expression of the genes of the CXCL cluster and their flanking genes (Figure 4A). In addition to the genes *CXCL8*, *CXCL6*, *CXCL1*, *ALB*, *MTHFD2L* and *AREG*, which are already expressed in undifferentiated THP-1 cells (Figure 2), we found the expression of *CXCL7* and *CXCL3*. Also in differentiated THP-1 cells *CXCL8* displays the highest expression within the investigated genomic region and showed 443-fold higher mRNA levels than *CXCL6*, 114-fold higher than *CXCL1*, 48-fold more than *CXCL7* and a 67-fold excess compared to *CXCL3*. Moreover, compared to undifferentiated cells, in PMA-differentiated THP-1 cells *CXCL8* is 33-times higher expressed. Detailed 8 h time course experiments in PMA-differentiated THP-1 cells showed that *CXCL8* (Figure 4B), *CXCL6* (Figure 4C) and *CXCL1* (Figure 4D) are primary 1,25(OH)_2_D_3_ target genes also in this cellular model. However, in these macrophage-like cells all three CXCL genes are less inducible than in undifferentiated THP-1 (monocyte-like) cells: even after 8 h stimulation with 1,25(OH)_2_D_3_ the induction of *CXCL8* is only 1.9-fold, that of *CXCL6* is 3.3-fold and and that of *CXCL1* is 3.0-fold. Furthermore, for all three genes a significant induction by 1,25(OH)_2_D_3_ was detected only after 2.5 to 3.5 h stimulation, i.e. clearly delayed compared to undifferentiated THP-1 cells. For comparison, in the same time course experiments the genes *CXCL7* and *CXCL3* showed no significant response to 1,25(OH)_2_D_3_ (Figure S2). Moreover, also in PMA-differentiated THP-1 cells the flanking genes *ALB*, *MTHFD2L* and *AREG* do not shown any early response to treatment with 1,25(OH)_2_D_3_ (data not shown).

Taken together, in differentiated THP-1 cells the CXCL cluster genes *CXCL8*, *CXCL6* and *CXCL1* are higher expressed than in undifferentiated cells. In addition, *CXCL7* and *CXCL3* expression is detected. However, also in differentiated cells *CXCL8*, *CXCL6* and *CXCL1* are the only primary 1,25(OH)_2_D_3_ targets within the CXCL cluster, but their inducibility by 1,25(OH)_2_D_3_ is reduced and delayed.

### VDR binding in PMA-differentiated THP-1 cells

**Figure 5:**
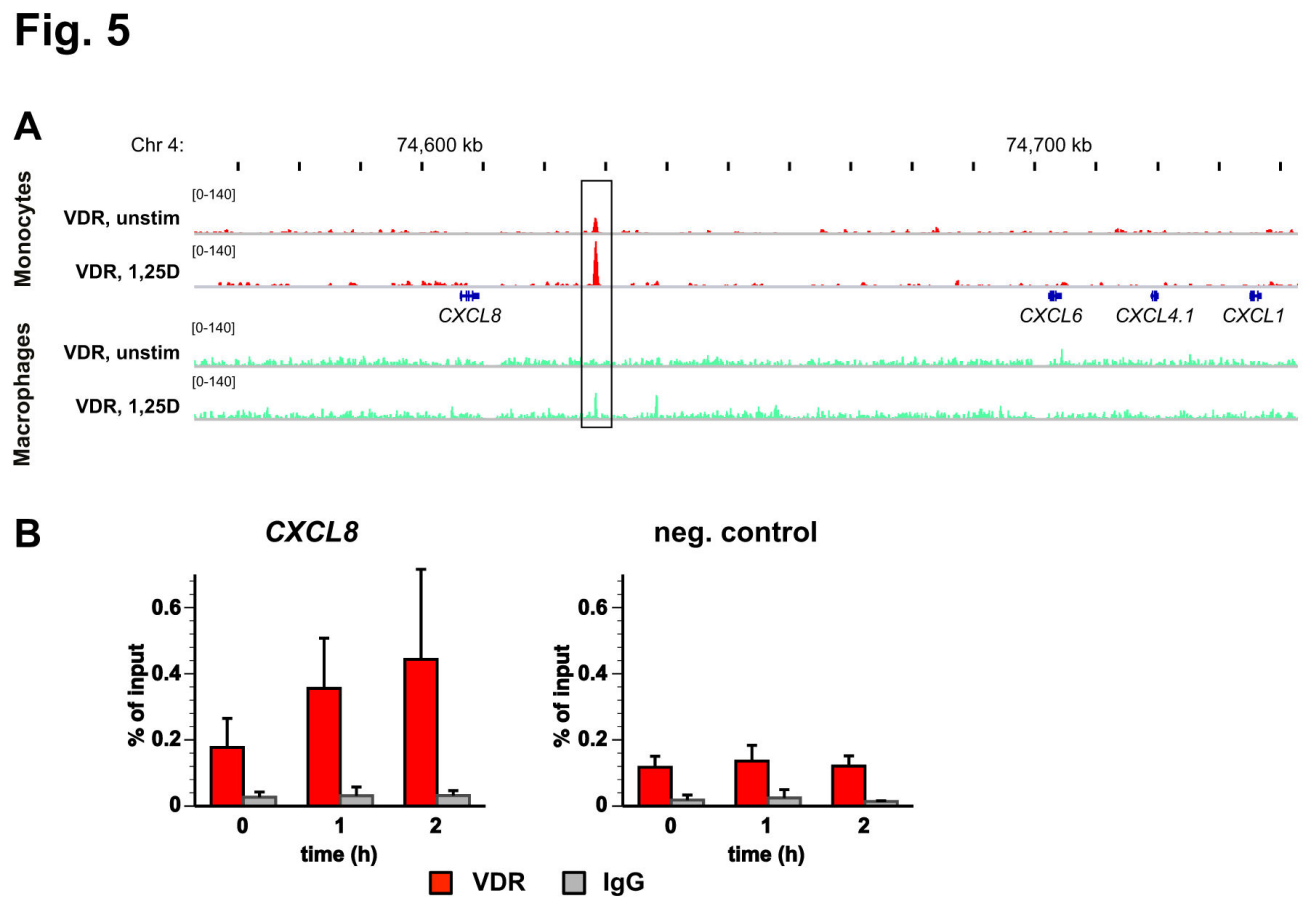
1,25(OH)_2_D_3_-dependent VDR association in PMA-differentiated THP-1 cells. A. The IGV browser was used to display the genomic region +/-150 kb around the VDR peak close to the *CXCL8* gene. The peak tracks show VDR ChIP-seq data obtained from undifferentiated THP-1 cells (red [32]) and from PMA-differentiated THP-1 cells (green). The gene structures are shown in blue. B. ChIP-qPCR was performed with chromatin samples obtained from PMA-differentiated THP-1 cells to determine VDR association (red) and unspecific IgG binding (grey) at the VDR binding site and a negative control region of chromosome 6. Cells were stimulated for 0, 1 and 2 h with 10 nM 1,25(OH)_2_D_3_ and chromatin was extracted. Columns represent the means of at least three independent experiments and the bars indicate standard deviations. Two-tailed Student’s t-tests were performed but could not determine significant 1,25(OH)_2_D_3_-induced VDR association in reference to untreated cells.

In order to investigate, whether a differentiation of THP-1 cells into macrophage-like cells modulates the VDR binding to the CXCL gene cluster, we performed ChIP-seq for VDR in PMA-differentiated THP-1 cells. In differentiated THP-1 cells we found VDR binding at the same location than in undifferentiated cells (Figure 5A). Moreover, by ChIP-qPCR in PMA-differentiated THP-1 cells we could confirm a ligand-dependent binding of VDR to this site (Figure 5B). Furthermore, we could not detect any additional significant VDR binding site within 3 Mb distance to the CXCL cluster, when the THP-1 cells were differentiated into macrophage-like cells (Figure 5A and data not shown).

In summary, in PMA-differentiated THP-1 cells the CXCL cluster is controlled by the same VDR binding site than in undifferentiated cells.

## Discussion

VDR ChIP-seq and microarray assays performed in undifferentiated THP-1 cells [32] indicated that the *CXCL8* gene may be a target of 1,25(OH)_2_D_3_ and its receptor VDR. Therefore, we investigated in this study the 1,25(OH)_2_D_3_ response of the whole CXCL gene cluster both in undifferentiated and PMA-differentiated THP-1 cells. We were able to confirm the primary response of *CXCL8* to 1,25(OH)_2_D_3_ in undifferentiated THP-1 cells and found the neighboring genes *CXCL6* and *CXCL1* to be primary VDR targets as well. In differentiated THP-1 cells the same three genes are also 1,25(OH)_2_D_3_ targets but, while they respond in undifferentiated cells already within 1 h after onset of stimulation, in differentiated cells their response was delayed by 1.5 to 2.5 h. Moreover, the prominent induction of *CXCL8* gene expression in undifferentiated cells is, dependent on the time of stimulation, 2- to 5-fold reduced in differentiated cells. However, the reduced responsiveness of *CXCL8* to 1,25(OH)_2_D_3_ in differentiated cells coincides with a 33-fold higher basal expression, so that in the latter cell type a stimulation with 1,25(OH)_2_D_3_ induces even a higher number of *de novo* synthesized *CXCL8* mRNA molecules than in undifferentiated cells. The same applies for the genes *CXCL6* and *CXCL1*, which showed an up to 2-times reduced inducibility by 1,25(OH)_2_D_3_, when THP-1 cells differentiate into macrophage-like cells, but increased their basal expression more than 2-fold.

VDR ChIP-seq analysis in undifferentiated and PMA-differentiated THP-1 cells suggests that the CXCL gene cluster is controlled by a single VDR binding site close to the *CXCL8* gene. The location of conserved, insulating CTCF binding sites suggest that the VDR binding site and all nine CXCL genes are located within the same chromosomal domain. FAIRE-seq data suggest that in undifferentiated THP-1 cells the genomic region around the genes *CXCL8*, *CXCL6* and *CXCL1* is far more accessible than the remaining CXCL cluster. Therefore, it is surprising that *CXCL4.1* gene, which is located between *CXCL6* and *CXCL1*, is not expressed in these cells. However, in tissues where *CXCL4.1* is expressed, it should be a VDR target gene. In contrast, although the genes *CXCL7* and *CXCL3* are expressed in differentiated THP-1 cells, they do not respond to stimulation with 1,25(OH)_2_D_3_. This suggests that PMA-differentiated THP-1 cells may use the CTCF sites 3 or 4, in order to loop to CTCF cite 2 (see Figure 1A), i.e. that shorter DNA loops may be formed in differentiated cells than in undifferentiated cells. 

Nevertheless, the VDR binding of the CXCL cluster belongs to a group of 165 genome-wide locations [51], for which a stimulation with 1,25(OH)_2_D_3_ results in a prominent opening of chromatin at the locus of VDR binding. Within this subset of VDR binding sites 66% carry a DR3-type response element within the sequence below the respective VDR peak summits [51]. This is a more than 2-times higher rate than the 31.7% reported for all genomic VDR binding sites in undifferentiated THP-1 cells [32]. Accordingly, we found below the summit of the VDR peak close to the *CXCL8* gene also a DR3-type response element. This suggests that the VDR binding site of the CXCL cluster can be distinguished from the majority of genomic locations of the VDR. We speculate that this site may represent a preferred contact point of the receptor with the genome, which may have been evolutionary selected. 

The facts that we observed i) a VDR binding site close to the *CXCL8* gene, ii) 1,25(OH)_2_D_3_-dependent chromatin opening at the latter site, iii) a DR3-type response element at this site indicating direct DNA binding of the VDR and iv) mRNA up-regulation of *CXCL8*, *CXCL6* and *CXCL1* suggest that the three genes are classical, up-regulated primary targets of 1,25(OH)_2_D_3_. This conclusion seems to contradict previous reports that 1,25(OH)_2_D_3_ represses *CXCL8* expression [18,20,53]. However, in these studies *CXCL8* gene expression [18,53] or *CXCL8* promoter activity [20] had been stimulated by the cytokines interferon-γ and tumor necrosis factor α, respectively, and by lipopolysaccharide, i.e. by stimuli for transcription factors, such as NF-κB, that strongly up-regulate the *CXCL8* gene. Moreover, two of these studies used primary monocytes [18,53] and one a melanoma cell line [20], i.e. cellular systems different from THP-1 cells. In contrast, a recent study in THP-1 cells, in which the cells were stimulated with a potent synthetic VDR ligand, is consistent with our finding that *CXCL8* gene expression is up-regulated [54]. Since VDR is known to negatively interfere with the activity of NF-κB [19] and other mediators of pro-inflammatory signaling pathways [55], the observations of Harant et al. [20], Di Rosa et al. [53] and Giulietti et al. [18] most likely represent repressing effects on a transcription factor activating *CXCL8* gene expression rather than any primary effect of VDR on the gene’s activity.

The above discussed points suggest that 1,25(OH)_2_D_3_ may have a dual effect on CXCL gene expression: a specific primary up-regulation via direct binding of VDR to the CXCL cluster locus and a more global secondary effect, by which all those genes are repressed that are early responding targets of NF-κB. Both effects depend on chromatin organization, i.e. the differentiation status of the cells, and external signals, such as paracrine effects of NF-κB-stimulating cytokines. In general, the actions of 1,25(OH)_2_D_3_ are summarized to be anti-inflammatory [56,57], to which the here reported up-regulation of three pro-inflammatory chemokines seem to be a contradiction. However, when the complex time- and signal-dependent inflammatory reaction is separated into its individual phases, it becomes clear that 1,25(OH)_2_D_3_ has a modulatory effect on all of them. This implies that in the early phase of inflammation, to which the up-regulation of chemo-attractant CXCL chemokines belongs, 1,25(OH)_2_D_3_ has a supporting role, while in the later phase a possible overreaction of the inflammatory response is controlled by 1,25(OH)_2_D_3_ via the repression of NF-κB. The potentiation of *CXCL8* expression by 1,25(OH)_2_D_3_ will lead to an initially more pronounced inflammatory reaction, which, dependent on the type of cancer, will either have an supporting or disadvantageous effect on cell survival [58]. In this way, our observation of the fast up-regulation of *CXCL8*, *CXCL6* and *CXCL1* by 1,25(OH)_2_D_3_ provides an additional aspect to the effects of the nuclear hormone on the immune response with impact on cancer immunology.

In conclusion, we found that both in undifferentiated and in PMA-differentiated THP-1 cells the genes *CXCL8*, *CXCL6* and *CXCL1* are primary targets of 1,25(OH)_2_D_3_ and its receptor VDR. Our observation implies a more differential view on the modulation of the inflammatory reaction by 1,25(OH)_2_D_3_ and provides further evidence for the impact of vitamin D in supporting the immune system in its fight against both microbes and cancer.

## Supporting Information

Figure S1 **Short- and long-term effects of HDAC inhibition on CXCL genes in undifferentiated THP-1 cells.** In THP-1 cells qPCR was performed to determine the relative changes of mRNA expression of the genes *CXCL8*, *CXCL6* and *CXCL1* in response to incubation with 100 nM 1,25(OH)_2_D_3_ (1,25D), 300 nM TsA, 3 µM SAHA and 1 mM VPA, alone or in combination, for 2.5 and 24 h. The data points represent the means of three independent experiments and the bars indicate standard deviations. Two-tailed Student’s t-tests were performed to determine the significance of the mRNA induction by the stimuli and the repression of the 1,25(OH)_2_D_3_ stimulation by HDAC inhibitors (* p < 0.05; ** p < 0.01; *** p < 0.001).
(TIF)

Figure S2 ***CXCL7* and *CXCL3* are no 1,25(OH)_2_D_3_ target genes cluster in PMA-differentiated THP-1 cells.** With samples obtained from PMA-differentiated THP-1 cells qPCR was performed to determine the change of expression of *CXCL7* (A) and *CXCL3* (B) in response to incubation with 10 nM 1,25(OH)_2_D_3_ over a time period of 8 h. Data points represent the means of at least three independent experiments and the bars indicate standard deviations. Two-tailed Student’s t-tests were performed to determine the significance of the mRNA induction by the stimuli (* p < 0.05).
(TIF)

Table S1 **Reverse transcription qPCR primers.**
(PDF)

Table S2 **ChIP-qPCR primers. All primers were designed using Oligo 4.0 software (National Biosciences).**
(PDF)
