# Supplementary figures and images for: Primary 1,25-Dihydroxyvitamin D3 Response of the Interleukin 8 Gene Cluster in Human Monocyte- and Macrophage-Like Cells
Source: PLoS One. 2013 Oct 21;8(10):e78170. doi: 10.1371/journal.pone.0078170 (PMC3824026; doi:10.1371/journal.pone.0078170)

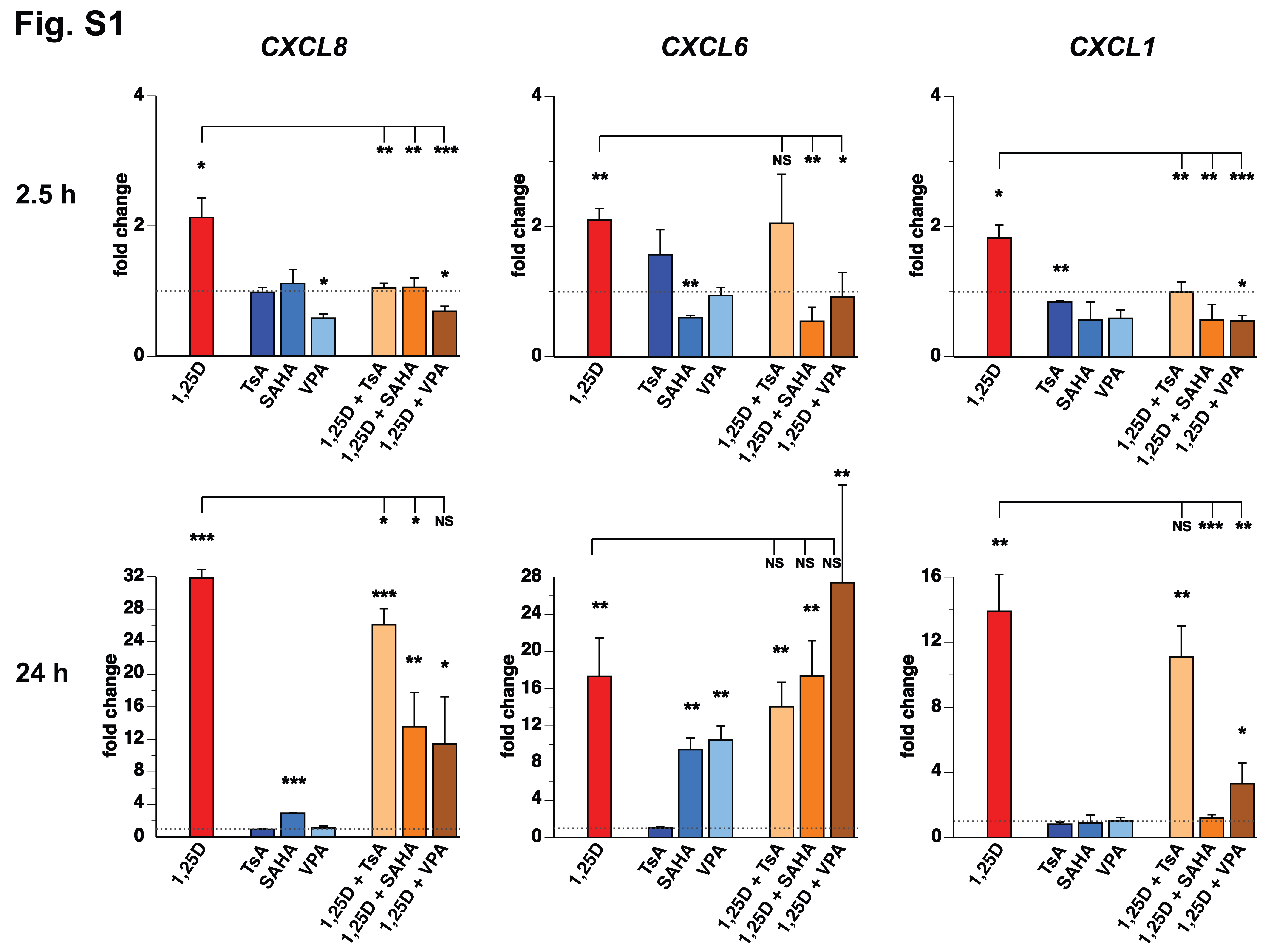

Supplement: Figure S1 — Short- and long-term effects of HDAC inhibition on CXCL genes in undifferentiated THP-1 cells. In THP-1 cells qPCR was performed to determine the relative changes of mRNA expression of the genes CXCL8, CXCL6 and CXCL1 in response to incubation with 100 nM 1,25(OH)2D3 (1,25D), 300 nM TsA, 3 µM SAHA and 1 mM VPA, alone or in combination, for 2.5 and 24 h. The data points represent the means of three independent experiments and the bars indicate standard deviations. Two-tailed Student’s t-tests were performed to determine the significance of the mRNA induction by the stimuli and the repression of the 1,25(OH)2D3 stimulation by HDAC inhibitors (* p < 0.05; ** p < 0.01; *** p < 0.001). (TIF) [file pone.0078170.s001.tif]

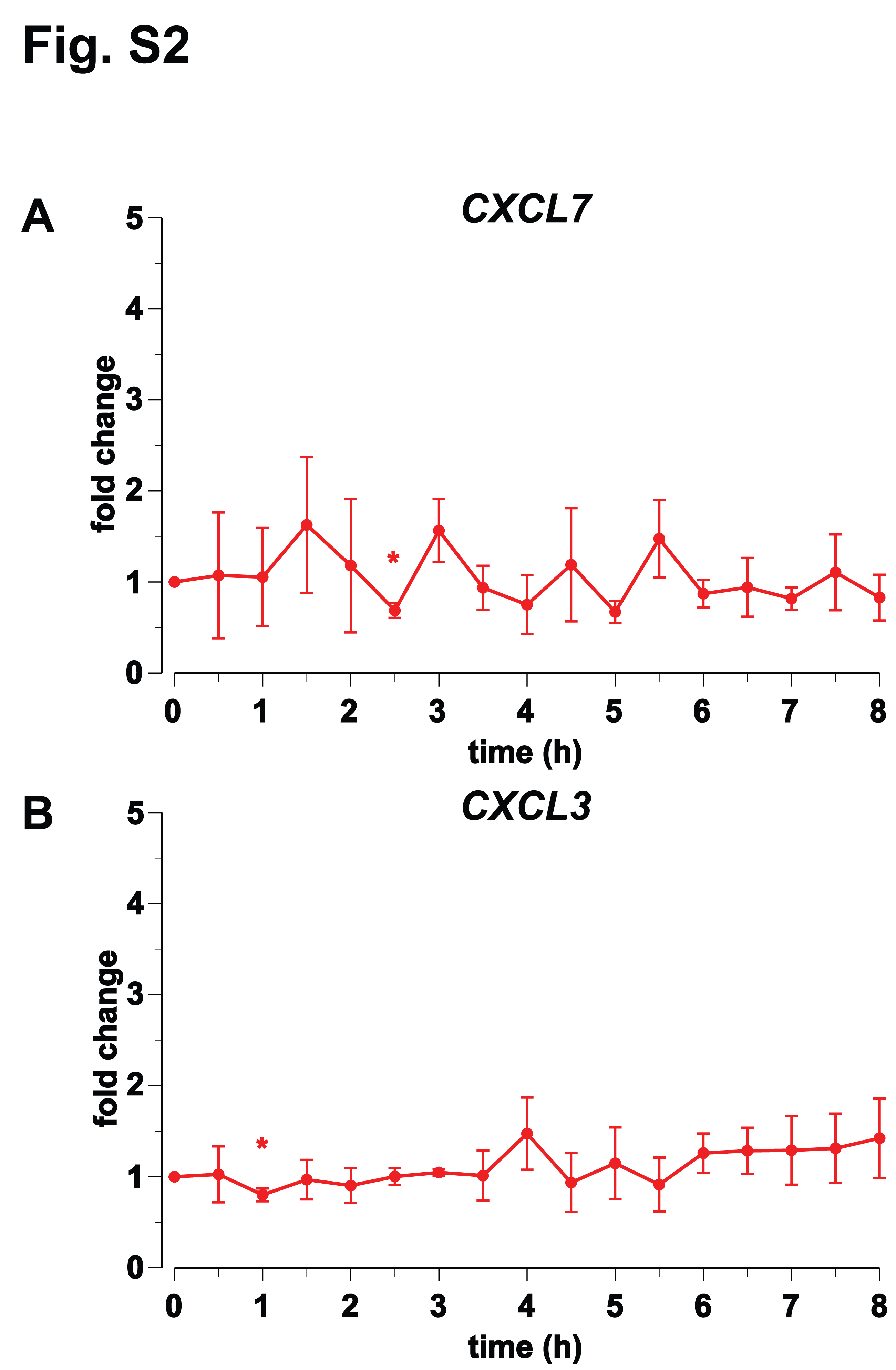

Supplement: Figure S2 — CXCL7 and CXCL3 are no 1,25(OH)2D3 target genes cluster in PMA-differentiated THP-1 cells. With samples obtained from PMA-differentiated THP-1 cells qPCR was performed to determine the change of expression of CXCL7 (A) and CXCL3 (B) in response to incubation with 10 nM 1,25(OH)2D3 over a time period of 8 h. Data points represent the means of at least three independent experiments and the bars indicate standard deviations. Two-tailed Student’s t-tests were performed to determine the significance of the mRNA induction by the stimuli (* p < 0.05). (TIF) [file pone.0078170.s002.tif]
