# Supplementary material for: Primary 1,25-Dihydroxyvitamin D3 Response of the Interleukin 8 Gene Cluster in Human Monocyte- and Macrophage-Like Cells
Source: PLoS One. 2013 Oct 21;8(10):e78170. doi: 10.1371/journal.pone.0078170 (PMC3824026; doi:10.1371/journal.pone.0078170)
Supplement: Table S1 — Reverse transcription qPCR primers. (PDF) [file pone.0078170.s003.pdf]

Table S1: Reverse transcription qPCR primers.

Gene	Fragment size (bp)	Annealing temperature (°C)	Primer sequences (5'-3')
AFM[1]	194	60	ATGTCTGTGGGGCACTTTTG<br>GTTCATAACCTTGCTCGTGTC
AFP[1]	173	56	GAAGAAGACAGGGAGACATTC<br>ACTGTTGCTGCCTTTGTTTGG
ALB[1]	134	58	GAAAGTACCCCAAGTGTCAAC<br>TCAGGACCACGGATAGATAG
AREG[1]	91	58	GGAGCCGACTATGACTACTC<br>CTACCTGTTCAACTCTGACTG
B2M[2,3]	246	60	GGCTATCCAGCGTACTCCAAA<br>CGGCAGGCATACTCATCTTTTT
CXCL1[1]	67	58	GATGCTGAACAGTGACAAATC<br>TCAGGAACAGCCACCAGTG
CXCL2[1]	279	58	CGAAAAGATGCTGAAAAATGGC<br>GGAGAGTGTGCAAGTAGATTC
CXCL3[1]	74	58	GATACTGAACAAGGGGAGCACG<br>CAGGAAGTGTCAATGATACG
CXCL4[1]	313	56	CGAGGCTCAGGAGTCATTG<br>GTGGCTATCAGTTGGGCAG
CXCL4.1[1]	141	56	CTGCCCAACTCATAGCCAC<br>TCACAGTTAGATTGAAAGTGC
CXCL5[1]	203	56	CGTTGCGTTTGTTTACAGAC<br>CAGTTTTCCTTGTTTCCACC
CXCL6[1]	146	58	CGAACCCTCTCTTGACCAC<br>AGCAGAGACAGGACCAGCG
CXCL7[1]	207	63	GCGAAAGGCAAAGAGGAAAG<br>CTTGATTCTGGGAGCATCTG
CXCL8[1]	168	58	CAGAGACAGCAGAGCACAC<br>AGCACTCCTTGGCAAAACTG
EPGN[1]	109/136	62	GACAGCACTGACCGAAGAG<br>CAGGCAAAGGTGTGAGAAC
EREG[1]	77	58	GAGGAGGATGGAGATGCTC<br>CTGCCTGTAGAAGATGGAAAC
GAPDH[2,3]	113	60	CATGAGAAGTATGACAACAGCCT<br>AGTCCTTCCACGATACCAAAGT
HPRTI[2,4]	94	60	TGACACTGGCAAAACAATGCA<br>GGTCCTTTTCACCAGCAAGCT
MTHFD2L[1]	136	62	CTCTGCTGTAGGTATTTGTAG<br>GTCTGGTAGTGGTAACTGAAC
RASSF6[1]	168	60	GCAAAGGAATGACACGCTGG<br>GCACTGGGGAGTCTGGTTC
RPLP0[1,2]	319	58-63	AGATGCAGCAGATCCGCAT<br>GTGGTGATACCTAAAGCCTG

[1] designed with Oligo 4.0 software (National Biosciences, Plymouth, MN, USA)
[2] reference gene

[3] Sequence obtained from PrimerBank (http://pga.mgh.harvard.edu/primerbank)
[4] see [59]
