# Supplementary material for: Primary 1,25-Dihydroxyvitamin D3 Response of the Interleukin 8 Gene Cluster in Human Monocyte- and Macrophage-Like Cells
Source: PLoS One. 2013 Oct 21;8(10):e78170. doi: 10.1371/journal.pone.0078170 (PMC3824026; doi:10.1371/journal.pone.0078170)
Supplement: Table S2 — ChIP-qPCR primers. All primers were designed using Oligo 4.0 software (National Biosciences). (PDF) [file pone.0078170.s004.pdf]

Table S2: ChIP-qPCR primers.

Genomic region	Fragment size (bp)	Annealing temperature (°C)	Primer sequences (5'-3')
CXCL8	93	58	GAATGTAGGTTGGGTGGAAC
VDR site			CAGGTGACTTGGACTCTATG
**CXCL cluster**	130	63	GGCACGGAATAACTGTCAAC
CTCF site 1			TCACTAGGTCAGGTGGCAG
**CXCL cluster**	100	63	GAGAGGAACAGGGGGAGTAG
CTCF site 2			CTGCCACTTATAGGTAGGATG
**CXCL cluster**	163	63	CAACACAGAGGACTCAAGAAC
CTCF site 3			CGCAGGAGAAACCAGAGGCA
**CXCL cluster**	174	63	CTCTCTTCTTTTAGCCAAGTG
CTCF site 4			TTCTAAGTCTGTCACAAGGTG
**CXCL cluster**	210	63	GAGGGAGAAATGTCTGTGAAG
CTCF site 5			CATAAGGAGGAGGACCAAAG
**CXCL cluster**	137	63	TGATGGCAGCATTGAGCAAG
CTCF site 6			GACACCAACCAATCCCACTG
**Chr 6 control region**	180	60	ATCACAGGGGTCAGCACATC
negative control			CGCAGCATTTGGGTTCACAC
